# HIV Testing Patterns among Black Men Who Have Sex with Men: A Qualitative Typology

**DOI:** 10.1371/journal.pone.0075382

**Published:** 2013-09-19

**Authors:** Sophia A. Hussen, Robert Stephenson, Carlos del Rio, Leo Wilton, Jermel Wallace, Darrell Wheeler, for the HPTN 061 Protocol Team

**Affiliations:** 1 Hubert Department of Global Health, Rollins School of Public Health of Emory University, Atlanta, Georgia, United States of America; 2 Department of Medicine, Division of Infectious Diseases, Emory University School of Medicine, Atlanta, Georgia, United States of America; 3 Department of Human Development, Binghamton University, Binghamton, New York, United States of America; 4 Graduate School of Social Work, Loyola University, Chicago, Illinois, United States of America; University of Missouri-Kansas City, United States of America

## Abstract

**Background:**

Black men who have sex with men (MSM) in the Southeastern United States are disproportionately affected by HIV. Black MSM are more likely to have unrecognized HIV infection, suggesting that testing may occur later and/or infrequently relative to current recommendations. The objective of this qualitative study was to explore the HIV testing behaviors of Black MSM in Atlanta, Georgia, who were participants in the HIV Prevention Trials Network Brothers Study (HPTN 061).

**Methods and Findings:**

We conducted 29 in-depth interviews and four focus groups with a community-recruited sample. Modified grounded theory methodologies were used to guide our inductive analysis, which yielded a typology comprised of four distinct HIV testing patterns. Participants could be categorized as: (1) Maintenance Testers, who tested regularly as part of routine self-care; (2) Risk-Based Testers, whose testing depended on relationship status or sexual behavior; (3) Convenience Testers, who tested irregularly depending on what testing opportunities arose; or (4) Test Avoiders, who tested infrequently and/or failed to follow up on results. We further characterized these groups with respect to age, socioeconomic factors, identity, stigma and healthcare access.

**Conclusions:**

Our findings highlight the heterogeneity of HIV testing patterns among Black MSM, and offer a framework for conceptualizing HIV testing in this group. Public health messaging must account for the diversity of Black MSM's experiences, and multiple testing approaches should be developed and utilized to maximize outreach to different types of testers.

## Introduction

In the United States, Black men who have sex with men (MSM) experience disproportionately high HIV infection rates despite similar or lower rates of risky sexual behavior relative to MSM of other races [1]–[3]. These disparities are exacerbated in the Southern United States, where the relative risk of living with HIV for Black MSM is estimated to be twice as high as what has been previously reported for nationwide samples [4]. Multiple explanations have been postulated to account for these disparities, but evidence suggests that one key factor is the higher rate of undiagnosed, and therefore uncontrolled, HIV infection among Black MSM, leading to increased viral transmission within their sexual networks [5]–[8]. Scale-up of testing efforts has been promoted as a critical first step towards making earlier diagnoses, facilitating linkage to care, and ultimately reducing the existing racial disparities in HIV incidence among MSM [9]–[11].

Despite higher rates of unrecognized HIV infections, the vast majority of Black MSM have been tested for HIV at some point in their lives [6], [9], [11], [12]. Furthermore, some studies have shown that, compared to White MSM, Black MSM are actually more likely to have been tested within the last year [13], [14]. One study of testing frequency found that Black MSM had shorter inter-test intervals than White MSM across a four-city sample of men attending STD clinics [15]. The fact that Black MSM have higher rates of undiagnosed HIV infection notwithstanding these findings suggests that, although likely to test for HIV on multiple occasions, the frequency of testing may still be sub-optimal. Another explanation for this seeming paradox is that these large-scale survey studies are somehow missing high-risk, low-frequency testers. Such sampling biases could be a result of recruitment strategies, as many of these studies were based on telephone surveys or recruitment in healthcare settings, approaches which would be less likely to access the most hard-to-reach members of this population.

Our current understanding of HIV testing practices among Black MSM is therefore insufficient to explain the racial disparity in undiagnosed infections [16]. Findings from one study demonstrated that different testing strategies (alternative venue testing, social networks strategies, and partner counseling and referral) were more effective for identifying undiagnosed HIV infection in varying subgroups of Black MSM, and that factors such as gay identity, age, and sexual risk predicted differential utilization of these testing programs [17]. An earlier study in Atlanta found that social and psychological factors, such as attending gay venues, experiences with homophobia, and investment in gay civil rights, were positive predictors of HIV testing within the past year [12]. These studies highlight the importance of considering diversity within Black MSM communities, and point to the need for multi-pronged testing promotion strategies targeting different subgroups. However, the range of HIV testing practices among Black MSM, and the mechanisms that create these varying practices, remain relatively understudied.

We therefore sought to enrich our understanding of the variation in HIV testing patterns among Black MSM, as a basis for improving our scientific knowledge of the mechanisms underlying undiagnosed infection. We conducted a multi-method qualitative study comprised of focus groups and in-depth interviews with Black MSM in Atlanta, Georgia. These two complementary qualitative methodologies were utilized to provide a multi-layered analysis of understudied dimensions related to HIV testing for Black MSM: focus groups were conducted to assess social norms and patterns, while in-depth interviews explored core conceptual domains related to personal experiences with healthcare and HIV testing. This type of multi-method triangulation is used as a way to increase the internal validity of qualitative studies [18], [19]. We focused on Atlanta because of the high prevalence of HIV in the Southeast, as well as the importance of considering geographically specific constructions of HIV, race, and sexuality that have been described in this region [20], [21]. The objectives of our analysis were to describe patterns of HIV testing behavior in our sample of Black MSM, and to better understand demographic, social and contextual factors with the potential to contribute to differences in testing behavior. A more in-depth understanding of HIV testing behaviors among Black MSM will be critical in order to improve public health messaging, increase uptake of testing, and ultimately decrease unrecognized infection in this group.

## Methods

The qualitative data from the present study were drawn from the HIV Prevention Trials Network Brothers Study (HPTN 061), a multi-site protocol designed to determine the feasibility and acceptability of a multi-component prevention intervention for Black MSM in six US cities. This analysis is based on data from two study sites within the Atlanta metropolitan area. The institutional review board at Emory University and the Grady Hospital Research Oversight Committee approved the study.

### Participants

Full details of the HPTN 061 protocol are described elsewhere [22]. For the (primarily quantitative) parent study, Black MSM were recruited directly from the community or referred by sexual network partners between September 2009 and September 2010. Recruitment methods included community outreach, engagement of community-based groups, advertising, and use of online strategies. Men were eligible to participate in the study if they: self-identified as a man or male at birth and as Black, African American, Caribbean Black, or multiethnic Black, were at least 18 years old, and reported at least one instance of unprotected anal intercourse with a man in the past six months. Men were ineligible if they were enrolled in any other HIV interventional study, if they had been a participant in an HIV vaccine trial, or if they were a community-recruited participant in a category (based on HIV status and risk behavior) that had already reached its enrollment cap. The study population included both HIV-negative and HIV-positive participants. From this larger group, a subset of participants also took part in a qualitative sub-study that is the basis for this analysis. Participant selection for the qualitative study initially followed a randomization scheme based on participant ID number; however, qualitative recruitment was later expanded to all participants regardless of ID number, until recruitment targets were reached. Participation in the qualitative portion was voluntary and concurrent with participation in the other parts of the HPTN 061 study.

### Data Collection

At the enrollment visit, study staff confirmed eligibility and obtained written informed consent. All participants then completed a demographic survey (which included age ranges but not exact ages), a behavioral assessment, and a social and sexual network questionnaire. Those who agreed to participate in the qualitative sub-study returned on a separate date to participate in either a focus group or an individual in-depth interview.

We conducted four focus group discussions. Each focus group was led by a trained Black, gay-identified, male facilitator who was a member of the study team and introduced himself as such at the beginning of the session. Focus groups were conducted in a private room in an office building. The core domains of the focus group guide were: (1) The lived experiences of participants in their local communities, (2) HIV/STI testing patterns and health care utilization, and (3) Stigma and social norms. The discussions lasted approximately 1.5 hours each and were digitally audio-recorded and then transcribed verbatim. In-depth interviews were later conducted with 31 participants. The semi-structured interview guide focused on: (1) Overall impressions about HPTN 061, (2) Health care utilization, (3) HIV/STI testing and counseling, (4) HPTN 061 service up-take, and (5) Stigma and discrimination. Two Black, male, gay-identified study team members conducted the interviews. Both interviewers had previous research experience and formal graduate education relating to health services research. The interviews were all conducted in a private room in an office building, averaged 45–60 minutes in length and were subsequently digitally audio-recorded and transcribed verbatim.

### Thematic Analysis

All analysis was done using MAXQDA10 (VERBI Software, Berlin, Germany), a qualitative data analysis software package. We utilized an inductive approach to our thematic analysis. Our first aim was to describe patterns of HIV testing behavior within our sample. As such, each interview transcript was read in detail by the first author, and all portions of text relevant to HIV testing were highlighted for subsequent review. Highlighted portions of text were then re-read for emergent sub-themes, yielding descriptions of the four testing categories discussed below (Maintenance Testers, Risk-Based Testers, Convenience Testers, and Test Avoiders). To ensure replicability, the transcripts were recoded at a separate sitting two weeks after the initial coding, to ensure that the assignment of testing categories to participants remained consistent, which they did.

Next, we analyzed the interview transcripts further within the four testing categories using modified grounded theory methodologies, which are useful for describing new conceptual frameworks to explain social processes [22]. We started with line-by-line analysis of selected, information-rich transcripts within each testing category. This generated more focused codes, which were then applied to subsequent transcripts, yielding more detailed information about contextual factors associated with each tester type. Finally, we turned to the focus group transcripts and coded these as well, in order to supplement our analysis with further information about the socio-contextual constructs discussed in the interviews.

## Findings

We conducted four focus groups with 30 participants (5–8 discussants per group) and individual in-depth interviews with 31 additional participants. Two of the interview transcripts were missing, leaving 29 interview transcripts available for analysis. The 59 Black MSM included in this qualitative analysis were primarily of low socioeconomic status (the majority reported annual household income less than $20,000), and they reported age ranges from 18–20 to 51–60 (see Table 1 for full demographic details). From our initial reading of the transcripts, it was clear that virtually all participants, regardless of their own HIV status, recognized the benefits and importance of HIV testing. Most participants described close friends or family members who were living with, or had died of HIV/AIDS, and many also provided narratives of learning their own HIV diagnosis. In spite of this generally high level of awareness, however, there was considerable variation in reported testing histories. Rather than the tester/non-tester dichotomy that is often described in the literature, what emerged was a more nuanced typology of testing patterns. The descriptions of the typology and the categories within it are derived entirely from the individual interview transcripts. Three interview transcripts did not provide sufficient detail about testing behaviors to be categorized, so they are omitted from this first part of the analysis. The remaining 26 interview transcripts could be divided into four categories based on the testing patterns that they described: the Maintenance Testers, the Risk-Based Testers, the Convenience Testers and the Test Avoiders.

**Table 1:** Demographic Characteristics of Qualitative Study Participants.

	Focus Groups	Interviews	Totals
**Age Range**			
18–20	2	2	4 (6.7%)
21–30	8	10	18 (30.5%)
31–40	6	7	13 (22.0%)
41–50	11	8	19 (32.2%)
51–60	3	2	5 (8.5%)
**Education (highest level completed)**			
Some high school	6	4	10 (16.9%)
High school grad	6	7	13 (22.0%)
Vocational training	1	1	2 (3.3%)
Some college	13	15	28 (47.5%)
College graduate	3	2	5 (8.5%)
Masters or other advanced degree	1	0	1 (1.7%)
**Household Income**			
<$5,000/yr	10	5	15 (25.4%)
$5,000–$9,999/yr	3	2	5 (8.5%)
$10,000–$19,999/yr	9	8	17 (28.8%)
$20,000–$29,999/yr	3	5	8 (13.6%)
$30,000–$39,999/yr	4	4	8 (13.6%)
$40,000–$49,999/yr	0	2	2 (3.4%)
$50,000–$59,999/yr	1	3	4 (6.8%)
**Healthcare Coverage**			
Yes	12	8	20 (33.9%)
No	18	21	39 (66.1%)

### Typology of HIV Testing Behaviors

#### Maintenance Testers (n = 9)

This group of participants described HIV testing as an integral part of their routine self-care. Most of them stated that they got tested every six months, which was cited as the optimal and expected frequency with which to get tested, as in the following example from an interview participant: “ It was just something that I would normally do. I normally get tested every six months ‘cause I knew once I became sexually active that was just the right thing to do—that’s how I was brought up, I guess.” The Maintenance Testers espoused an internal locus of control with regards to their healthcare and repeatedly cited the importance of advocating for their own health and having up to date information about their bodies.

I know it’s important for you to know your body, you have to live with you every day. Every day, every minute, every hour, every second, you have to live with you so I feel as if you need to take care of yourself. You need to know yourself. You need to know what’s going on in your body. (*Interview Participant*)

With regards to HIV, Maintenance Testers were aware of the availability of effective treatment, and therefore thought it was better to know one’s status in order to get into care and start antiretroviral therapy. The same participant went on to say:

Sometimes I’m scared to get tested for HIV but, I know that it’s very deadly and if I have it then, well I figure if I get it then it’s better for me to find out sooner than later because if I find out sooner than maybe I can help it. So it wouldn’t completely take over my body and I won’t have, it would be too late you know…better safe than sorry.

#### Risk-Based Testers (n = 7)

This group did not test as frequently or regularly as the Maintenance Testers, but did so when they perceived their risk to increase to a level that would warrant an HIV test. Sometimes this risk perception changed after a known exposure to HIV, as below:

Last time I was tested before that was like oh, it must have been about five years and then the time before that was probably five years, cause I lost a lover to the virus in 1989 and I got tested then and I was getting tested regularly for the next couple of years after that, but then after that, I kind of stopped…I was thinking about, I was thinking about it being overdue, but it wasn’t any real rush. (*Interview Participant*)

In other cases, testing was prompted by a change in relationship status or changes in frequency of sexual behavior:

*Interviewer*: [Before this study], when had you been tested?

*Participant*: The last time was about like two years before, because I wasn’t too sexually active. I was kind of you know, cause I was living in New York at the time…and I didn’t really do nothing because I was helping my family out…I keep to myself when I’m at home. And you know, came back here …being sexually active, but it was with my partner that I dated for awhile and yeah.

*Interviewer*: Okay so how many times where you tested before?

*Participant*: Before that I was tested like every six months.(*Interview participant*)

This quote highlights the important caveat that these categories are not static—this participant would have been classified a Maintenance Tester in the past, but stopped testing regularly when he became either less sexually active or monogamous. Others similarly endorsed the belief that HIV testing was primarily for single people, as opposed to those in stable relationships.

#### Convenience Testers (n = 4)

All of the Convenience Testers mentioned free testing as a benefit to enrolling in the HPTN 061 study. One interview participant stated, “That was one of the main reasons [I decided to participate], I was told it was a place that was private and you know a good place to come and get tested.” Within this group, cost was repeatedly brought up as a barrier/facilitator to HIV testing. Comparing HIV to other sexually transmitted disease (STD) testing, another interview participant noted: “You know what, I haven’t been tested for STDs as common as I have been for HIV. Like that’s the only test everybody gives out for free.” In addition to seeking free testing, this group also utilized testing opportunities offered in their neighborhood or at an event that they were attending for other reasons. At times, these services built on pre-existing social networks or cultural affirmation:

Well the first time I ever got tested was in [city name]. And the only reason why I got tested is cause a friend of mine worked for an organization called [name]. Based out of [second city name], and they came into town and they brought a test with them to my house, so that was the first test I ever got was at my house and when I passed it, I was like okay this is not even painful, so this is something that really if you’re sexually active and you’re not practicing safe sex that you should do. At least you know, even if you, I mean you are practicing safe sex, but if you mess up one time, you still don’t know, so. Then after that test, I went to [city, state] gay pride and I took a test in the park, I was negative. (*Interview participant*)

From these excerpts, we can see that HIV testing is acceptable to this group when it is provided free of charge, and in a convenient location where they feel comfortable.

#### Test Avoiders (n = 6)

The final category of participants that emerged from our interview data was the Test Avoiders. None of these individuals were completely avoiding testing, as they all tested voluntarily as a part of participation in this study. However, half of them were being tested for the first time, and the others had been tested before but admitted that they frequently failed to follow up for test results. Fear of positive results was mentioned as a specific barrier in several of these transcripts.

*Interviewer*: Tell me why you didn't go back for your results?

*Participant*: It’s basically stupidity I would say because I could've, I could've went back but, it just didn't fit, the fear of the results being positive. The fear of hearing that, that's the main thing nobody want to hear that but, you know when you, you know it is what it is. You have to accept it for what it, you know, I did it with the Brothers project and I found out [that I was HIV positive], I was hurt but its life you have to deal with it to the best of your abilities. It's not going to be something that you just going to be like who you know, it’s going to be so much trouble that you can just think about cause I’m still like blown over it but…

*Interviewer*: So why did you decide to just go ahead and get that test results this time?

*Participant*: Because I knew I'd be in this. I knew I'd been gay or in this homosexual life going on 13 years now and I'm in the process of transferring myself from men to transgender woman, so I knew you know what I'm saying? I knew I had to face reality sooner or later.(*Interview participant*)

As this passage shows, the participant knew the risks of her own sexual behavior, and knew that testing was indicated. In her case, the high level of perceived risk and fear of having to deal with positive results caused her to delay definitive knowledge of her HIV status.

### Social-Contextual Attributes of the Testing Categories

Once the four testing categories were described, we analyzed demographic, social and contextual attributes of each group in order to better understand why these varied patterns exist, and to potentially form a basis for public health messaging. Descriptions of experiences with regard to age, identity, stigma, and healthcare access were especially salient, with some variation seen between the testing groups (summarized in Table 2). The findings that follow were derived from all 29 individual interview transcripts, the four focus group transcripts (n = 30 participants), and the demographic survey data.

**Table 2:** Characteristics of Interview Participants in the Four Testing Categories.

	Maintenance	Risk-Based	Convenience	Avoider
Number of participants	9	7	4	6
Age (median age range)	21–30	31–40	41–50	21–30
Income (median income range)	$20,000–29,999	$20,000–29,999	$20,000–29,999	$20,000–29,999
Education (proportion with “some college” or more)	9/9 (100%)	3/7 (43%)	3/4 (75%)	3/6 (50%)
Identity	Majority gay-identified	Split between gay and bisexual self-identification	Majority bisexual-identified	Majority gay-identified and/or transgender
Salience of race vs sexuality	Sexuality > Race	Race = Sexuality	Race > Sexuality	Sexuality > Race
Stigma	++	+	+	+++
Healthcare access*	8/9 with full or partial access	6/7 with full or partial access	2/4 with partial access, none with full access	3/6 with full or partial access

* Healthcare access definitions: **Full = **answered “yes” to having healthcare coverage and/or described a relationship with a primary medical doctor.

**Partial = **described routinely accessing care, even if through emergency departments.

**None = **described no access to healthcare.

+reported some experiences with stigma and discrimination, ++ reported frequent stigma and discrimination, +++reported frequent and severe stigma and discrimination.

#### Age and other demographic variables

We compared demographic characteristics (age, income, and education) of the four groups. The most notable inter-group differences were seen in the age distribution. The Maintenance Testers were relatively young (median age range 21–30). The Test Avoiders were also young on average, with a similar median age range. Risk-Based and Convenience testers, in contrast, tended to be relatively older (median age range 31–40 and 41–50, respectively). Several participants discussed age and intergenerational differences in HIV risk. Although many of the younger men who were interviewed described themselves as having very responsible behavior with respect to HIV testing, older participants specifically stated that HIV prevention efforts needed to target youth.

I really don’t know numbers or statistics but, but in my, in my experience a lot of us are still having unprotected sex, you know, with the younger generation coming behind me, I just think that more money needs to go into outreach and education. Can’t stress that enough. We could probably never have too much of that. (*Focus group participant*)

When we examined levels of educational attainment within the groups, we found that the Maintenance Testers described more postsecondary education than the others, in large part because over half of the Maintenance Testers were current college students. Two additional Maintenance Testers had already graduated from college. None of the interview participants in any of the other testing categories were college graduates, although many had some college education. Only one other individual was a current college student (a Test Avoider who was between 41 and 50 years of age and enrolled in a community college). In spite of the between-group differences in educational level, however, there were no noticeable differences in income distribution across the groups.

#### Identity and Stigma

Experiences of stigmatization and discrimination varied considerably, as did self-assigned identity labels. The Maintenance Testers generally described themselves as openly gay, in both the demographic survey and the qualitative interviews. Related to this, they were also very pro-social and involved in a variety of organized activities in their communities, including but not limited to involvement with organizations centered around gay identity. This group also experienced frequent discrimination based on sexuality, primarily in work and school settings.

The Test Avoiders also described themselves as openly gay, including two participants who were transgendered (male-to-female). The most severe and frequent bullying and victimization was described by Test Avoiders, several of whom stated that for them, sexuality-based discrimination was a daily occurrence. One example follows:

I mean it happens every day. You may be walking down the street and you might see a guy that you may think is like really cute, but he's straight and so the first thing that comes out of his mouth is probably fag or sissy… I mean, I was walking down the street one day and someone had said fag. I think it was during Gay Pride weekend or something like that, but that's common. (*Interview participant*)

On the other hand, all of the men in the Convenience Testers group and most of the men in the Risk-Based group described themselves as not easily being “clockable” [recognizable] as gay, and as a result described relatively few experiences of overt discrimination based on their sexual preference, as one Risk-Based Tester illustrated: “I really wasn’t that discriminated against that much, cause I ain’t let anybody know. I was deep in the closet. I was deep in the closet, usually the only people that knew were people that was doing it just like me.” (*Interview participant*)

These men generally described themselves as having very masculine external presentations, and they tended to endorse more traditional masculinity norms and beliefs. Several of these men expressed the sentiment that other gay men should also act more masculine when interacting with mainstream society, and limit more feminine behaviors to gay venues or to home.

In contrast, experiences of race-based stigma and discrimination were more commonly described by the Convenience and Risk-Based Testers. As a result, these men advocated for prevention strategies that focused more on racial solidarity and manhood as opposed to a gay identity. An interview participant in the Risk-Based group, for example, stated: “We could be tested for the same thing, but at the same time we can be Black with different sexual orientations, different walks of life, but we still are Black brothers, you know what I’m saying? In other words, expand the program to all Black men.”

The differences between participants' experiences with identity and stigma may have been largely a function of differences in age. Several participants discussed ways in which the experience of being Black and gay had changed over time. Sometimes this intergenerational difference was described as increasing acceptance of same-sex identities, which was seen as favorable to the younger generations:

I just turned forty-eight…looking down, you know, looking at these other children growing up, I feel kind of proud for me, because I marched to make it [happen]. It was unheard of for you to come out at twelve or thirteen years old when I was growing up in my lifetime. You know when I was young, you had to stay buried in the closet. You know so, after I marched and stuff to make gay rights and gay pride day and all this, what y’all have in [Atlanta] I did it in [another city], you know to make that gay rights at the forefront. It makes me proud to know that I have done that, you know, as far as being in the trenches and stuff. (*Focus group participant*)

Others, however, saw the increased openness about sexuality as making the younger generations more vulnerable to homophobic attacks.

Looking at our youth, I see them at being more vulnerable at being discriminated than when I was coming up. They’re more out you know, out of the closet, and…when they see young black men you know, dressing feminine you know, like a female or having makeup done or wearing revealing clothing, they get, I mean, the feedback from a lot of the younger, other generation, it’s horrifying you know…call ‘em faggots. Call ‘em gay. Want to kick their butts. They pick fights with ‘em. They groups up and try to you know, to infuriate that person, make that person feel inferior, cause they crowds around ‘em and they say all these horrible things about ‘em you know and it’s more like to embarrass them you know, put ‘em to shame. (*Focus group participant*)

#### Healthcare Access

The demographic questionnaire included a dichotomous (yes/no) question about whether or not participants had healthcare coverage. Based on their answers to this question, a very low proportion of the participants (8/29 interview participants, 20/59 of total qualitative sample) had healthcare coverage, and no patterns could be discerned relating healthcare coverage to the different categories in the testing typology. However, qualitative analysis of the interview transcripts showed that healthcare access was more complex than what was captured by the single dichotomous (yes/no) question about healthcare coverage on the survey, and that the survey question actually underestimated interactions with the healthcare system in several cases. One of the participants in the Risk-Based Tester group, who answered “no” to the survey question, illustrated such a scenario:

*Interviewer*: Now you said you go to the hospital when you’re sick with asthma, who do you rely on for your health care needs?

*Participant*: Nobody. I don’t have no kind of health care plan, nothing like that.

*Interviewer*: You have no health insurance?

Participant: No.

*Interviewer*: Okay so when you do get sick, what hospital do you go to?

*Participant*: [name] hospital.

*Interviewer*: Okay and what insurance do you use when you go there?

*Participant*: I don’t use insurance. I just go. Like everybody else I know.

*Interviewer*: Okay, okay. So you don’t have a health care provider?

*Participant*: Doctor [name] at the [name] County Health Department.

*Interviewer*: Oh okay. What’s your relationship like with your doctor?

*Participant*: It’s excellent. I’ve been knowing her since I was like 21 years old. Every time she sees my face it’s like she be knowing me, it’s like I’ve been knowing her for so long you know it’s like, we like this, we, I mean she’s a good doctor to me.I’ve been knowing her for like what, since I was like 21. I’m 33 years old now. (*Interview participant*)

We therefore re-conceptualized healthcare access, using both survey and interview data, as “full”, “partial” or “none”. Participants who answered “yes” to having healthcare coverage on the survey and/or described a relationship with a primary medical doctor were categorized as having full access to healthcare. Some of this discrepancy was explained by the fact that HIV-positive individuals in Atlanta can access regular healthcare through Ryan White-funded clinics, even if they are uninsured. Participants who did not have coverage but described routinely accessing care through emergency departments or walk-in clinics, were defined as having “partial” access to care. Those who stated they did not have healthcare coverage on the survey *and* denied regularly accessing healthcare in the interview were categorized as having no access. Comparing healthcare access across testing categories, the Maintenance and Risk-Based Testers were more likely to have full or partial degree of access to healthcare, when compared to the Convenience Testers and Test Avoiders, who were more likely to describe having no access to healthcare relative to the other two groups.

## Discussion

Our typology of HIV testing behaviors highlights intra-group diversity and variation in testing patterns among Black MSM, who are often conceptualized as a homogenous risk group in epidemiological studies. The Maintenance Testers tested frequently and routinely, and represent a previously under-reported category of Black MSM. Most prior studies of repeat HIV testing in MSM were conducted before 2003, when regular, repeated HIV testing became the standard national recommendation [23], [24]. Although the majority of this early work conceptualized repeat testing as a marker for high-risk behavior, a few studies also described a subgroup of young, health-conscious regular testers who were similar to the Maintenance Testers in our typology [25], [26]. Our findings take this concept further by highlighting additional attributes of the Maintenance Testers. In addition to their young age, they were also more educated and had more regular access to the health care system relative to the other groups. The Maintenance Testers also described internalization of norms favoring testing every six months, suggesting that public health messaging has effectively communicated the most recent testing guidelines to this group.

The Risk-Based Testers varied their testing practices depending on sexual activity and relationship status. Their attitudes toward testing are consistent with older public health messaging, in which regular testing was not the paradigm of choice. On the whole, this group was older than the Maintenance Testers, and they may have internalized these previous testing recommendations as the norms on which they still based their testing behaviors. Another important finding was that Risk-based Testers described testing less frequently when in stable relationships. This pattern is consistent with prior studies demonstrating that MSM in primary relationships test at lower rates than the general MSM population [27].

The transcripts from the Convenience Testers highlighted the role of structural barriers such as cost of testing, location, and access to healthcare. These findings are consistent with previous studies in which convenience and accessibility of testing services were been described as facilitating HIV testing [28]. Notably, none of the participants in this group had consistent access to regular health care. This association between inadequate healthcare coverage and irregular testing is consistent with a recent study of Black men in Georgia, which found that health insurance coverage not only facilitated initial HIV testing, but also increased testing frequency [29].

Finally, the Test Avoiders were most likely to be affected by fear, which is a salient construct in the literature on barriers and facilitators of HIV testing. Fear in this context includes fear of being stigmatized if an HIV test is positive, and/or fear of illness and death [30]–[33]. Consistent with our findings, fear has also been related to a desire to remain in denial about a likely positive result [34]. Fear of a positive result has also been described as a reason for not following up on HIV testing results [35], another pattern that was described by our participants.

In addition to defining and describing the four testing categories, our analysis also yielded interesting findings with respect to psychosocial factors including gay identity and experiences with stigma. Both identity and stigma seemed to be related to age and intergenerational differences in our sample, with younger men being more likely to report gay identity and experiences of sexuality-related discrimination. Very few studies have previously examined HIV testing in the context of these types of psychosocial factors. In our study, all of the Convenience Testers and many of the Risk-Based Testers highlighted their masculinity and described themselves as having an external presentation that was not discernibly gay. One prior study found that heterosexual self-presentation was negatively related to HIV testing in MSM, presumably due to the association between HIV testing and being “outed” as gay [36]. Although that study was conducted in a predominantly White sample, masculinity norms within the Black community have also been demonstrated to be relevant to HIV prevention behaviors among Black men [37]–[40]. On the other end of the spectrum, the Test Avoiders were more likely to describe themselves as gay and/or transgendered, and were the group in which the highest levels of sexuality-based stigma and discrimination were described. Recent work in South Africa showed similar findings among MSM, with feminine gender expression and sexual orientation-based stigma being positively associated with fear of HIV testing, perhaps due to discrimination in healthcare settings [41]. Interestingly, experiences of homophobia were positive predictors of HIV testing in a prior Atlanta-based study [12]. It may be that in the latter study, the homophobia was not necessarily internalized, but experienced more frequently due to higher levels of gay identification among HIV testers. The Maintenance Testers in our study similarly described high levels of gay identification, frequent discrimination, and frequent testing.

Our typology has potential implications for public health messaging and testing promotion strategies. The Maintenance Testers, whose reported testing patterns reflect current national guidelines, represent a group on which to model strengths-based approaches to HIV testing promotion. In contrast, the Risk-Based Testers might benefit from prevention strategies designed to update their knowledge of HIV testing recommendations. As the Risk-Based Testers also described decreased testing frequency in the context of stable partnerships, an additional potentially useful strategy for targeting this group may be couples-based HIV testing, which has been shown to be theoretically acceptable and appealing to Black MSM [42], [43]. This is particularly important given that the majority of HIV transmissions among MSM in the U.S. are likely occurring between main partners [44]. In addition to improving healthcare coverage, potential interventions to target the Convenience Testers include venue-based and in-home HIV testing, both of which have demonstrated high acceptability to Black MSM [45], [46]. The cost of the recently approved over-the-counter home HIV testing kit may be prohibitive for many, however, interventions to make home testing more affordable and accessible could have a significant impact for this subgroup of testers[47]. For the Test Avoiders, utilization of rapid testing is critical to ensure that those who do get HIV testing are notified of their results before being lost to follow up. Given their severe experiences with stigma and discrimination, culturally competent health services will also be particularly important for this group.

Our findings related to psychosocial constructs may also inform strategies for public health messaging. Messaging targeting older groups of Black MSM may be more effective if there is a focus on Black brotherhood and masculine imagery. These Black MSM, unlike the younger Maintenance Testers, may be less inclined to respond to public health campaigns that specifically cater to men who define themselves as gay. Gay identity also has implications for testing in the healthcare setting, where men who do not disclose their same-sex behavior to health care providers are often less likely to undergo HIV testing [48]–[50]. Men in the Risk-Based and Convenience group who may be less likely to identify as gay, or to be perceived as gay by their providers may not be offered testing as frequently as other MSM. This points to the need for providers to be proactive and encourage HIV testing for all men.

The qualitative and exploratory nature of this work precludes generalizability, but points to several important areas for further scientific inquiry. Future studies should be conducted in order to determine whether or not the proposed typology is applicable in other geographic settings and in larger, or different, study populations. The income and educational attainment described by our participants was consistent with that of the larger HPTN 061 cohort, but reflects a lower socioeconomic status group relative to the general U.S. population, the Black U.S. population, and the Black population of Atlanta [51], [52]. Future studies that include different socioeconomic strata may find additional or different types of testers than what we found in this sample. Our findings also suggest that future studies of HIV testing might benefit from further elaboration and operationalization of the typology, in order to create more informative HIV testing outcomes for large-scale survey studies. Finally, subsequent studies should aim to look more broadly at influences on HIV testing, and consider including measurements of psychosocial factors such as stigma and identity in addition to traditional demographic indicators.

### Limitations

This paper represents a secondary analysis of qualitative data. Since our research questions were not the only focus of the original parent study, and all of the analysis was conducted after data collection was completed, we may not have reached theoretical saturation. That is, more nuances or patterns could have emerged if we had interviewed more men with specific attention to HIV testing patterns, identity, stigma, and health care access. The participants' descriptions of their HIV testing histories were not corroborated with actual medical records and were thus subject to recall and social desirability biases. Our sample included both HIV-positive and HIV-negative participants, who may recall their experiences with testing in ways that differ from one another. Finally, this sample was recruited from a study that required participants to test for HIV. Those who truly wanted to avoid testing completely would be unlikely to participate in HPTN 061, biasing our sample towards pro-testing attitudes.

### Conclusions

Our analysis provides a useful new framework for understanding HIV testing among Black MSM, emphasizing the need to move beyond dichotomous conceptualizations of testing in order to achieve the frequent, regular testing that is recommended by current national guidelines. Our findings highlight the need for culturally relevant public health messaging and HIV testing initiatives that reflect the diversity of Black MSM's experiences and testing preferences, in order to maximize efficacy and relevance to the target population. Multi-pronged testing promotion strategies, including innovative modalities such as couples-based testing and decentralization of testing venues, as well as messaging targeting different age groups and identity beliefs, must be developed and utilized, in order to reach and benefit a wider range of Black MSM.
